# Pilates and obesity: A practical and promising option

**DOI:** 10.3934/publichealth.2026037

**Published:** 2026-06-24

**Authors:** Alexiοs Batrakoulis, Marilyn Gilyana, Mary Ann Rumplasch

**Affiliations:** 1 Department of Life Sciences, European University Cyprus, Nicosia, Cyprus; 2 Department of Physical Education and Sport Science, Democritus University of Thrace, Komotini, Greece; 3 College of Sport Science, University of Kalba, Kalba, Sharjah, UAE; 4 Blood Cancer United, Washington, DC, USA

**Keywords:** exercise prescription, overweight, physiological adaptations, psychological adaptations

## Abstract

Pilates is a type of mind-body fitness that may lead to significant improvements in body composition, physical fitness, cardiometabolic health, and mental health. However, these findings are supported by very limited and often mixed or scarce evidence. In general, Pilates training may promote exercise engagement of people with larger bodies in a physically and mentally comfortable environment, thereby promoting their health, performance, and well-being. Research suggests that integrating Pilates training as a component of a comprehensive, tailored, supervised exercise program for individuals with excess body weight and adiposity may offer a pleasurable and pain-free training alternative in practical settings. In this review, we synthesize the scientific evidence and a real-world Mat Pilates workout routine for obese individuals.


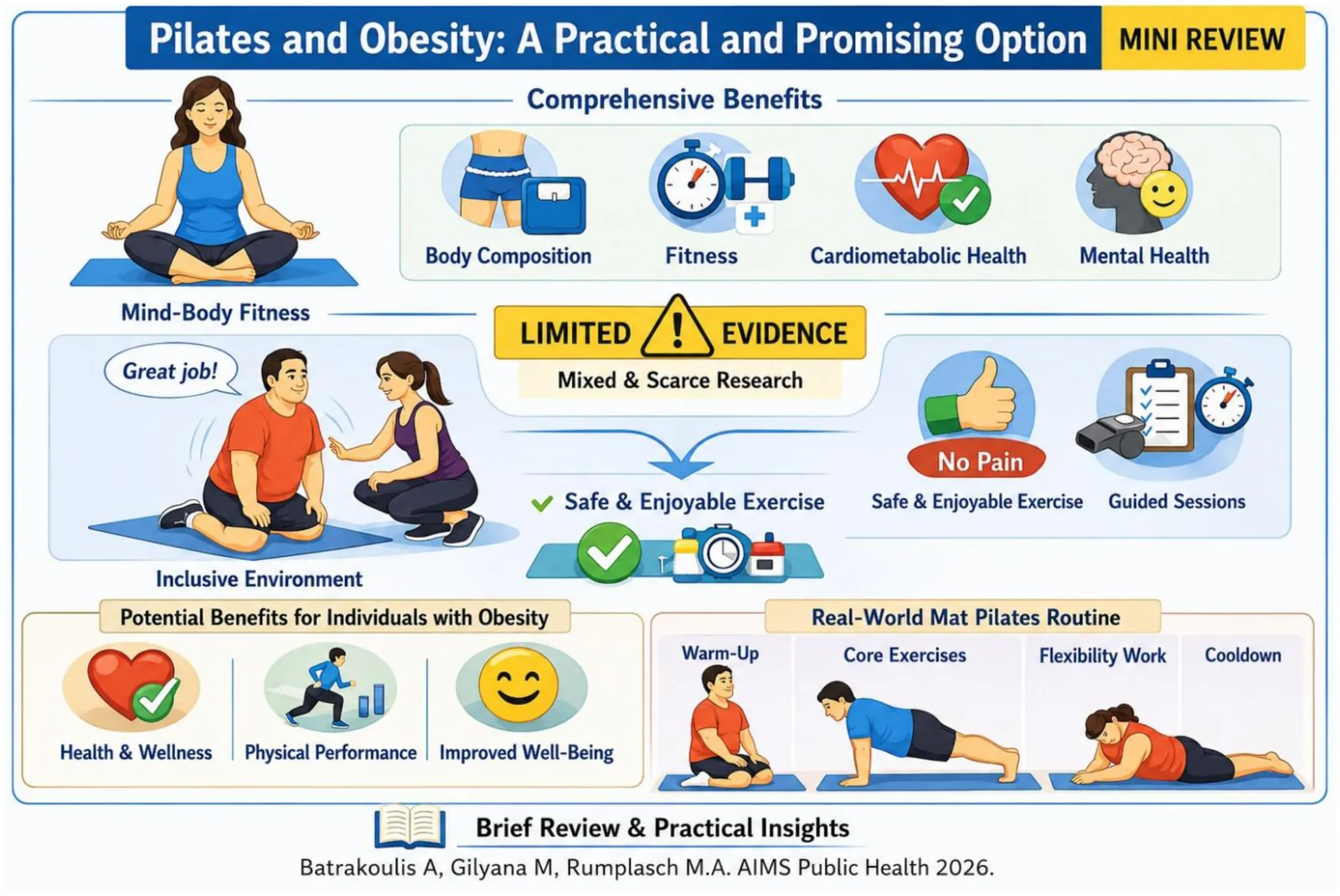


**Graphical abstract**

## 1. Introduction

### 1.1. The obesity epidemic

Obesity represents a significant global public health challenge, with well-documented links to considerable physical, psychological, and social burdens [1]. The adverse effects of this condition on daily functioning and health-related quality of life are well-documented, and there is a strong epidemiological link between the condition and an increased risk of metabolic, cardiovascular, and respiratory diseases, as well as several forms of cancer [2]. The prevalence of being overweight and obesity has increased markedly over decades, placing considerable pressure on healthcare systems and public health resources [3]. Furthermore, excess body weight and adiposity, particularly when combined with physical inactivity, have been identified as significant contributors to poor metabolic health and the development of obesity-related comorbidities [4],[5].

Obesity now recognized as a chronic, relapsing, and multifactorial disease influenced by biological, behavioral, psychosocial, and environmental factors [1]. The health implications of excess body mass extend beyond the mere presence of obesity, encompassing adipose tissue dysfunction, chronic low-grade inflammation, and impaired metabolic regulation. Collectively, these factors contribute to an elevated cardiometabolic risk profile [3]. Individuals with a larger body size frequently exhibit insufficient levels of physical activity, reduced functional capacity, cardiometabolic complications, impaired mental well-being, weight stigma, and body image dissatisfaction [5].

These considerations underscore the need to identify efficacious, viable, and sustainable interventions for overweight or obese adults. While weight reduction remains a significant treatment objective, substantial health benefits may also be achieved through improvements in cardiometabolic health, physical fitness, functional capacity, and psychological well-being, even in the absence of substantial weight loss [3]. Consequently, lifestyle-based strategies, notably structured physical activity and exercise, have garnered mounting attention as accessible and non-pharmacological approaches for enhancing health outcomes in this demographic [6]. Our primary objective of this review is to provide a summary of the evidence on the topic of Pilates in adults with obesity. Moreover, an example of a comprehensive, supervised, tailored, mat Pilates training program is provided to support practitioners working with individuals suffering from obesity in the field.

### 1.2. Literature search

#### 1.2.1. Methods

Literature for this narrative review was identified through searches of PubMed/MEDLINE and Google Scholar databases using combinations of keywords related to overweight, obese, obesity, and Pilates. Priority was given to English-language peer-reviewed articles published from inception to January 2026. A comprehensive search of reference lists from articles and other related resources was conducted to identify any additional relevant articles. Human studies involving adults any age with BMI ≥ 25 kg/m² and no diagnosed comorbidities, symptoms of non-communicable disease, or eating disorders were included. Interventions had to involve any form of Pilates and report psychophysiological outcomes. Studies were excluded if they included mixed samples with overweight/obesity plus other chronic diseases, used multicomponent programs that prevented isolation of Pilates effects, did not assess any psychophysiological outcomes, were published in languages other than English, or were not fully peer reviewed.

#### 1.2.2. Results

The electronic search returned 37 articles. After screening titles, abstracts, and full texts, 19 eligible studies were included in this review that were published from 2006 to 2025. The age of the participants ranged from 30 to 60 years, and the sample size varied from 28 to 108 participants, predominantly overweight or obese females across all studies. The duration of training studies ranged from 4 to 24 weeks, with exercise session frequency varying from three to five times per week. Quantitative methods were employed in the study design. Most articles reported on studies that assigned supervision and were conducted in a laboratory setting.

## 2. Evidence-based foundations of Pilates in larger bodies

### 2.1. Mind-body fitness for larger bodies

Studies have documented the supporting role of various mind-body fitness modalities, including the Pilates method, in the prevention, management, and treatment of obesity [7]. Beyond the scope of Pilates, other complementary therapies that focus on the mind-body connection have also been shown to be effective for adults suffering from obesity [8]. Yoga has the strongest obesity-specific evidence and may be particularly suitable because it can be adapted to reduced fitness, joint discomfort, balance limitations, and low exercise confidence [9],[10]. Mindfulness-based interventions may also serve as feasible adjuncts, given their low resource requirements and potential to improve eating-related behaviors and engagement in physical activity among overweight or obese adults [11]. Tai chi and qigong represent additional promising options, especially for individuals who may find conventional exercise difficult, as these modalities emphasize slow controlled movement, breathing, postural control, and body awareness [8]. Although obesity-specific evidence for tai chi and qigong remains limited, pilot studies have reported favorable adherence, high completion rates, and no serious adverse events [12],[13]. Overall, non-Pilates mind–body therapies appear feasible in this population; however, larger and longer-term trials are needed to establish their sustainability, acceptability, and comparative effectiveness [8],[12],[13].

### 2.2. Pilates training for larger bodies

Pilates, created by Joseph Pilates in the 1920s, is a mind-body exercise approach that emphasizes precise movement, posture alignment, and controlled breathing [14]. It can be practiced on the floor with or without props or through specialized equipment exercises that resemble conventional resistance training [7],[8]. This method aims to enhance core strength, flexibility, muscular endurance, coordination, balance, and body awareness while promoting a heightened mind-body connection that supports functional ability and self-confidence, particularly in individuals with higher body weight [14],[15].

Specialized Pilates equipment, including devices like the Reformer, Cadillac, Chair, and Barrel, employs spring-based resistance to provide adjustable, low-impact loading. Exercises on these machines are performed with controlled motions that engage muscles effectively without causing fatigue, making the practice accessible for beginners and individuals with physical limitations [7]. Evidence increasingly supports Pilates as an effective intervention not only for athletes and normal-weight individuals but also for overweight or obese people. In previously inactive populations, Pilates has been associated with favorable changes in body composition, metabolic health, blood pressure, and psychological well-being [16],[17]. These benefits are likely due to the integrative nature of Pilates, which combines fitness components into structured, supervised sessions [7].

Most studies targeting overweight or obese participants have examined mat-based exercises in supervised, laboratory-like settings, with limited data on equipment-based approaches in these populations [16]. Overall, Pilates may appear as safe and feasible for individuals with excess body weight, improving physical fitness and body composition with minimal risk of injury [17]. However, the evidence is inconclusive as available data are limited, often mixed or scarce, and of low methodological quality, as reported in the relevant trials in the literature. More specifically, studies have produced inconsistent results, leading to a lack of consensus and comparative studies in this area [16]. Taking this into account, more robust research is needed to clarify its impact on cardiometabolic markers and mental health outcomes among overweight/obese individuals [16]. Figure 1 summarizes the potential health and fitness benefits of Pilates for overweight or obese people.

**Figure 1.**
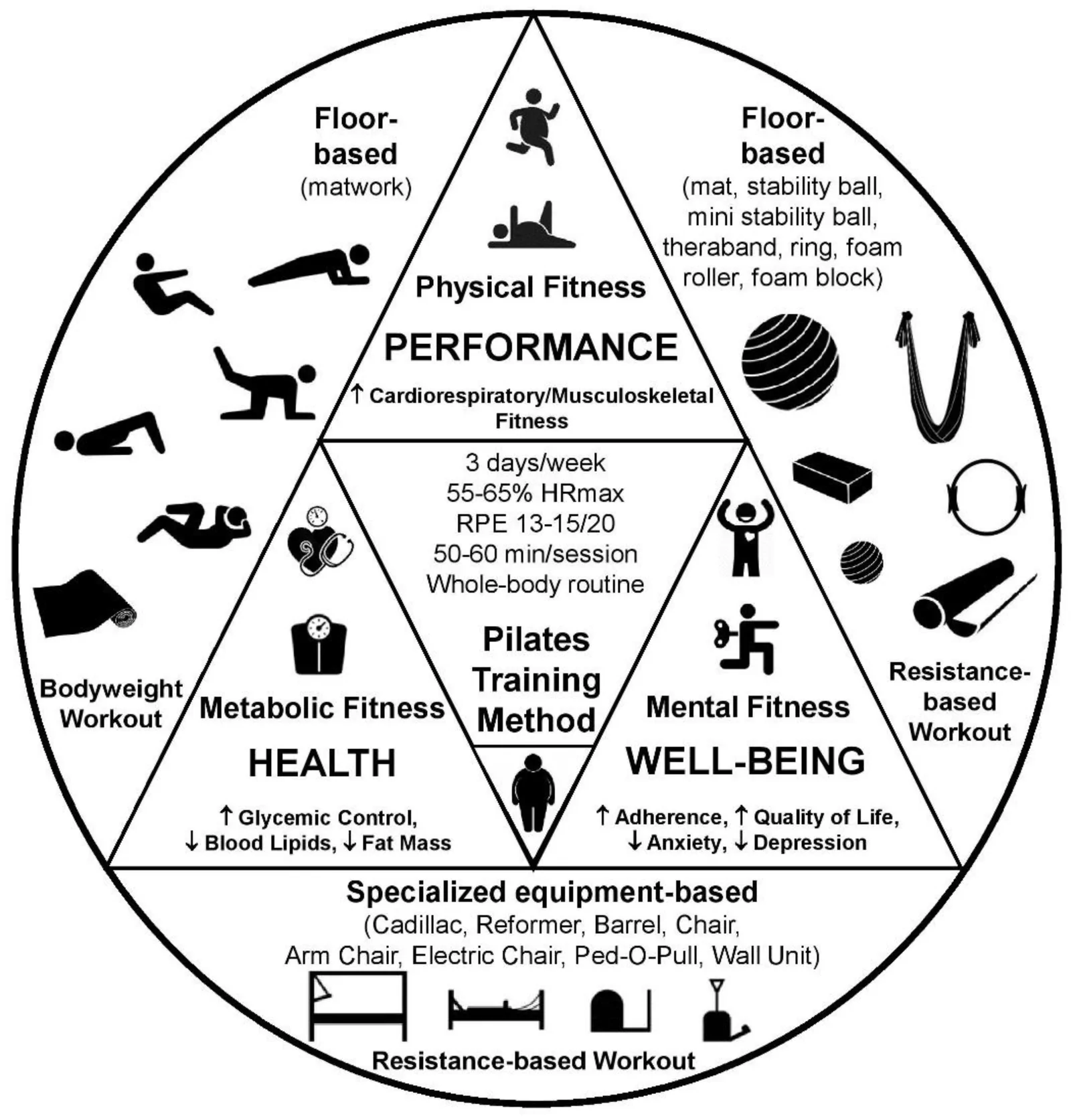
Summary of potential health and fitness benefits of Pilates training in overweight/obese people. HRmax, maximum heart rate; RPE, rating of perceived exertion.

### 2.3. Physiological adaptations

#### 2.3.1. Body composition

Research into Pilates in overweight and obese individuals has focused primarily on anthropometric and body composition outcomes, whereas evidence for psychological and broader clinical effects remains comparatively limited [17]. The literature suggests that Pilates may engender modest improvements in body weight, body mass index, body fat percentage, and waist circumference. However, these findings should be interpreted with caution because the evidence base is relatively small and methodologically heterogeneous [16],[17]. Conversely, there is a paucity of evidence to support meaningful effects on lean body mass or bone metabolism [16]. Consequently, while Pilates appears to be a promising strategy for body composition management in sedentary adults with overweight or obesity, its effectiveness cannot be considered firmly established. The proposed mechanisms are plausible, as Pilates involves repeated body-weight resistance movements and may contribute to energy expenditure when performed regularly, such as three 60-minute sessions per week [16]–[18]. However, the extent to which these physiological demands result in clinically meaningful fat loss remains to be substantiated through larger and more rigorous controlled trials [19].

#### 2.3.2. Physical fitness

Pilates is principally a neuromuscular training modality, not a cardiovascular exercise, but it may improve cardiorespiratory fitness in inactive adults [7]. The program emphasizes repetitive resistance-based movements targeting the core without inducing exhaustion. This approach has been shown to enhance endurance, stability, flexibility, balance, and posture while lowering injury risk [20]. These benefits are particularly salient for individuals with a BMI categorizing them as overweight or obese, who often face postural problems, functional limitations, and higher injury risk [21]. The literature also demonstrates the positive effects of intervention on physical–functional performance in normal-weight and overweight older adults [22]–[24]. However, research on flexibility, aerobic capacity, and broader musculoskeletal fitness outcomes remains limited, highlighting the need for further studies to clarify Pilates' effectiveness compared with other neuromuscular exercise types [16].

#### 2.3.3. Cardiometabolic health

Individuals with a BMI categorized as overweight or obese frequently exhibit elevated cardiovascular and metabolic risk factors [25], thus emphasizing the need to examine whether Pilates can enhance physiological outcomes pertinent to disease prevention and management. This increased risk has been linked to physical inactivity, excess visceral adiposity, chronic inflammation, insulin resistance, and oxidative stress [26]. Despite the fact that a number of exercise modalities have been shown to have a beneficial effect on glucose regulation, lipid profile, blood pressure, and physical fitness in individuals with unhealthy body mass [27], the evidence specific to Pilates remains limited and less conclusive. Studies have suggested the existence of potential benefits; however, the findings from cardiometabolic outcomes have proven to be inconsistent, and the number of robust randomized controlled trials remains limited [16]. In particular, there is an absence of compelling evidence to determine whether Pilates produces clinically meaningful improvements in cardiovascular risk factors that are comparable to those observed with more established exercise modalities. Consequently, the status of Pilates is that of a potentially beneficial, albeit preliminary, option for cardiometabolic health in overweight or obese adults, pending stronger comparative and long-term evidence [16].

### 2.4. Psychological adaptations

#### 2.4.1. Mental health

Psychological disorders are common among overweight or obese individuals [28], making it important to explore the effects of exercise, including Pilates, on mental health. While regular exercise is known to improve psychological distress, mood, and vitality, evidence for its impact on anxiety and depression in this population is limited [28]. Research on Pilates is particularly scarce, though it has shown benefits for mood and quality of life in older adults with abnormal BMI [24], and one study found improvements in anxiety and depression following both Pilates and aerobic training in adults with excess weight [29]. Given the lack of robust evidence, the role of Pilates in enhancing mental health status in this demographic remains unclear [30],[31].

#### 2.4.2. Health-related quality of life

Pilates has been demonstrated to engender a number of clinically relevant benefits for overweight or obese adults. As a low-impact and adaptable exercise modality, it may be particularly suitable for individuals with reduced fitness, excess joint loading, or low movement confidence [16]. Evidence from studies conducted on adults with excessive weight suggests that Pilates can improve health-related quality of life and other psychological well-being markers, thereby indicating potential benefits that extend beyond physical conditioning alone [29],[32]. Moreover, the evidence specific to obesity suggests favorable effects on body weight, body mass index, and body fat percentage, indicating that Pilates may contribute to improved body composition and overall health status [16],[17]. The findings of this study also suggest improvements in physical fitness, postural control, muscular endurance, body awareness, and function, which may facilitate daily activities and perceived well-being [16]. The findings, when considered as a whole, lend support to the notion that Pilates can be utilized as a practical adjunct to obesity management, with the potential to engender benefits for physical health and psychosocial outcomes that are relevant to long-term quality of life [16],[17],[29],[32].

#### 2.4.3. Adherence

Most Pilates studies have reported low rates of attrition; however, this phenomenon remains unelucidated in the literature. The potential for high adherence to Pilates training interventions is noteworthy, given the well-documented challenges associated with a lack of time, which is a significant barrier to exercise engagement [33]. This observation is important given that overweight and obese individuals frequently exhibit high attrition and poor compliance with other exercise modalities [34]. Nevertheless, the body of evidence is inadequate to substantiate the long-term implications of Pilates on adherence. Considering that muscle-strengthening activities are recommended twice weekly for overweight and obese adults [19], Pilates may serve as a suitable alternative resistance-based modality for this population.

Researchers should focus on the role of various forms of Pilates training with respect to training configuration and potential mechanisms behind favorable effects in psychophysiological indices. Large-scale, long-term (>6 months) studies with high methodological interventions should be conducted for larger individuals [16].

#### 2.4.4. Limitations and future directions

The studies included in this narrative review are subject to several important limitations that should be acknowledged. First, a significant number of studies were conducted with relatively small sample sizes, which reduces statistical power and limits the generalizability of the findings. Second, the duration of the interventions was often limited, which hinders the ability to ascertain whether the observed benefits can be maintained over an extended period. Third, considerable heterogeneity was evident across studies in participant characteristics, Pilates protocols, comparator groups, and outcome measures, which complicates direct comparison of results. Concerns regarding the methodology of the study were also identified, including the inadequate reporting of randomization procedures, limited blinding of outcome assessment, incomplete adherence data, and variable control of potential confounders. Furthermore, most researchers have focused on specific subgroups, particularly women, thereby reducing the generalizability of the findings. It is recommended that researchers place greater emphasis on the execution of larger, well-designed randomized controlled trials that employ standardized Pilates interventions. These trials should be complemented by extended follow-up periods, a more diverse population base, and a comprehensive evaluation of cardiometabolic, functional, and psychosocial outcomes. Last, it is interesting to note that Pilates training was not reported among the top health and fitness trends within the exercise community worldwide, although exercise for weight management has been documented as one of the most popular trends by practitioners [35]. This finding serves to corroborate the absence of any concrete evidence pertaining to the association between Pilates and obesity, underscoring the need for additional, high-quality research in this field.

## 3. Tailored applications of Pilates for larger bodies

### 3.1. Special safety and training considerations

Given the prevalence of poor functional capacity and common musculoskeletal health disorders among larger individuals [21], it is imperative that exercise professionals possess the expertise to address these limitations in a real-world gym setting. Addressing these challenges necessitates a comprehensive approach, entailing sensitivity training for professionals, personalized exercise programs, psychological support, and the establishment of an inclusive environment that fosters participation without judgment. Exercise professionals frequently encounter physical limitations when designing and instructing Pilates training sessions for their clients. The most common of these limitations is the inability to fit into machines, the inability to get up from the floor, the inability to stand up from a seated position, the full range of motion to bend over, and the inability to move around the equipment quickly and easily [36]–[38]. Therefore, Pilates, as a method that is centered on the development of core strength, flexibility, and control, may offer a positive experience for individuals with obesity. Particularly, mat Pilates training programs offer a supportive and safe alternative, making the practice accessible for individuals facing challenges with balance, mobility, or weight-bearing movements. On the other hand, evidence regarding the effectiveness of specialized equipment-based Pilates interventions in sedentary populations who are overweight or obese is limited. The following provides an example of a comprehensive, supervised, tailored, mat Pilates training program.

### 3.2. Mat Pilates: 30-Minute workout for clients with obesity

#### 3.2.1. Overview

This Pilates mat workout is designed for individuals with obesity, providing a gentle, safe, and effective approach to developing core strength. The routine highlights slow and controlled movements, minimal position changes, and comfortable postures. Most exercises are done lying on the back or side, which helps avoid stress on the wrists, knees, and neck. Emphasizing core stability, mindful breathing, flexible spine movement, and gentle strengthening, this workout aims to boost confidence and body awareness without requiring high-impact or demanding positions. The exercises are straightforward, steady, and can be adjusted to fit individual comfort needs.

The selected Pilates exercises were chosen not only on the basis of feasibility, safety, and adaptability for individuals with excess body weight and adiposity, but also because the literature suggests that Pilates-based training may improve core stability, flexibility, balance, functional performance, and psychological well-being. Nevertheless, it should be acknowledged that the evidence does not establish the superiority of any specific Pilates exercise combination, and thus the selected protocol should be considered a practical and theory-informed approach rather than a definitively optimized intervention.

This adapted Pilates mat workout for people with obesity aims to i) enhance core strength and stability, which is essential for addressing back and joint discomfort that may result from excess weight; ii) increase mobility, focusing on gentle movement in the shoulders, hips, and spine to promote greater range of motion; iii) strengthen the lower body, targeting muscles that support the hips and glutes, contributing to overall stability; and iv) develop body awareness, encouraging mindful movement and improved control over the body. The utilization of the following three props is optional, but may be advantageous for the execution of this Pilates mat workout. The props include a Pilates ring, a light to moderate resistance band, a yoga block, a foam cushion, a pillow, a towel, or a blanket.

#### 3.2.2. Exercises

**Warm-Up (5 Minutes)**

*Breathing and Centering*:

1. Lie on your back with knees bent hip-distance apart and feet on the mat.

2. Maintain a natural curve in your lower back. This is known as neutral spine and is unique to everyone.

3. Place hands on sides of ribcage and inhale through your nose for a count of five. As you inhale, feel the sides of your body expand. Exhale through pursed lips for a count of five, feel the sides of your body coming to a rest. Imagine your abdomen inflating and deflating like a balloon.

4. Repeat rounds of breathing for one minute.

Primary Muscles Worked: Diaphragm and core muscles.

*Pelvic Tilt*:

1. Lie on your back with knees bent hip-distance apart and feet on the mat, neutral spine. Place arms alongside your body with palms down.

2. Exhale, draw your navel toward your spine, and gently press your lower back into the mat to flatten the spine, tucking the tailbone. This is a posterior tilt.

3. Pause for a moment.

4. Inhale, return to the neutral position, creating the small arch in the lower back.

5. Pause for a moment.

6. Repeat the tuck and arch movement for 30–60 seconds.

Note: Keep the movement small and isolated to the pelvis and lower back.

Primary Muscles Worked: Core, pelvic floor, gluteal, and lower back muscles.

Note: Conscious pelvic floor contraction should be practiced during the working phases, and conscious relaxation during the rest phases.

*Scapular Protraction/Retraction*:

1. Lie on your back with knees bent hip-distance apart and feet on the mat, neutral spine. Extend your arms straight up toward the ceiling, with palms facing each other (imagine you're holding a large beach ball), wrists in line with the shoulders. To help increase body awareness and muscle activation during the movement, you can hold a Pilates ring with both hands, palms pressing into the outer pads.

2. Exhale, gently reach your hands and arms upward toward the ceiling, sliding your shoulder blades away from one another (protraction). Keep your arms long throughout the movement.

3. Pause for a moment.

4. Inhale, gently squeeze your shoulder blades together (retraction). Imagine you're cracking an egg between your shoulder blades. Avoid arching your lower back.

5. Pause for a moment.

6. Repeat rounds of scapular protraction/retraction for 30–60 seconds.

Primary Muscles Worked: Scapula and shoulder girdle stabilizers.

*Head Nod*:

1. Lie on your back with knees bent hip-distance apart and feet on the mat, neutral spine. Place arms alongside your body with palms down.

2. Exhale, slowly nod your chin toward your chest, think of making a “double chin”. Avoid any movement of the shoulders.

3. Pause for a moment.

4. Inhale, return to the starting position.

5. Repeat nods for 30–60 seconds.

Primary Muscles Worked: Sternocleidomastoid, scalene, longus colli, and rectus capitis lateralis muscles.

**Main Workout (20 Minutes)**

*AB Prep*:

1. Lie on your back with knees bent hip-distance apart and feet on the mat, neutral spine. Place arms alongside your body with palms down.

2. Inhale, gently nod your chin toward the chest to lengthen the back of your neck.

3. Exhale, contract your abdominal muscles (gently draw your navel toward your spine) and lift your head, neck, shoulder blades, and arms off the mat, sliding the rib cage down toward the pelvis and the arms reaching toward your feet, level with your shoulders. Keep your gaze toward your knees. Maintain neutral spine.

4. Inhale, pause for a moment keeping the core engaged.

5. Exhale, slowly lengthen back to the mat, using the core muscles to control the descent.

6. Repeat for 8-10 repetitions.

Modification: To alleviate neck tension, place hands behind the head to support the weight with elbows facing outward.

Primary Muscles Worked: Core, diaphragm, and pelvic floor muscles.

Note: Conscious pelvic floor contraction should be practiced during the working phases, and conscious relaxation during the rest phases.

*Shoulder Bridge*:

1. Lie on your back with knees bent hip-distance apart (or slightly wider if it improves balance and stability) and feet on the mat, neutral spine. Place arms alongside your body with palms down. To engage your inner thigh muscles (adductors) and ensure proper knee alignment with your hips, you can squeeze a Pilates ring or yoga block between your knees.

2. Exhale, gently tilt the pelvis to curl the tailbone upward, and lift the hips off the mat one vertebra at a time.

3. Inhale, pause for a moment holding the bridge position, squeezing the glutes. Maintain a long, stable line from the shoulders to the knees and avoid overarching the back and placing pressure on the neck.

4. Exhale, slowly roll your spine back down to the mat, one vertebra at a time, returning to the starting position.

5. Repeat for 5 repetitions.

Modification: If spinal articulation is not possible, hinge at the hip and keep a neutral spine as you lift and lower.

Primary Muscles Worked: Glutes, hamstrings, core, erector spinae, and pelvic floor muscles.

Note: Conscious pelvic floor contraction should be practiced during the working phases, and conscious relaxation during the rest phases.

*Toe Taps*:

1. Lie on your back with your legs in a “tabletop” position (knees bent at a 90-degree angle, stacked over the hips, lower legs parallel to the floor, ankles in line with the knees), imprinted spine (lower back pressed toward/into the mat). Place arms alongside your body with palms down.

2. Exhale, keeping your core engaged and lower back pressed into the mat, slowly lower one foot tapping the toe to the floor while keeping the 90-degree bend in the knee constant. If you cannot tap the floor, move your leg as far as you can while maintaining a stable pelvis and spine.

3. Inhale, return the leg to the tabletop position.

4. Repeat alternating sides 5–8 times each.

Modification: Rather than lifting the legs into the tabletop position, start with feet flat on the mat, hip-width apart, and knees bent. From this position, carefully lift one foot one to two inches off the mat, then return it to the floor with control.

Primary Muscles Worked: Core, pelvic floor, and hip flexor muscles.

Note: Conscious pelvic floor contraction should be practiced during the working phases, and conscious relaxation during the rest phases.

*Clamshell*:

1. Lie on your side with knees bent at approximately 90 degrees, hips stacked one on top of the other, feet in line with the spine, top hand on mat for balance, neutral spine (a small, folded towel under the waist can help maintain neutral alignment). If lying on the side is uncomfortable, use a pillow to support the head.

2. Exhale, keep your feet touching as you slowly open the top leg like a clamshell. Imagine a string pulling your top knee upward. Keep your pelvis still and lift only as high as you can without your hips rolling backward. To add resistance, loop a light to moderate resistance band around your thighs, just above the knees.

3. Pause for a moment.

4. Inhale, slowly lower your leg to the starting position.

5. Repeat 8-10 repetitions then switch sides.

Modification: If hips/knees feel tight, move the legs forward as in a “chair” position.

Primary Muscles Worked: Gluteus medius (most prominent) and gluteus minimus muscles.

*Side Lying Leg Lift*:

1. Lie on your side with bottom knee bent for stability and top leg straight in line with pelvis, hips stacked one on top of the other, top hand on mat for balance, neutral spine (a small, folded towel under the waist can help maintain neutral alignment). If lying on the side is uncomfortable, use a pillow to support the head.

2. Exhale, point your top foot and slowly lift your top leg about 6–12 inches. Focus on lifting with your outer hip, not your lower back.

3. Inhale, flex foot keeping toes facing forward and slowly lower your leg.

4. Repeat 6–8 times then switch sides.

Modification: To make the exercise easier, shorten the lever by keeping the top knee bent while lifting the leg.

Primary Muscles Worked: Gluteus medius (most prominent) and gluteus minimus muscles.

*Half Rollback*:

1. Sit tall just back of sit bones, knees bent with feet on the mat and hip-distance apart, hands behind thighs, neck relaxed, gaze straight ahead. If hip flexors are tight, sit on a folded blanket to elevate hips.

2. Exhale, draw core muscles in and roll your pelvis backward just a few degrees (think tiny tailbone tuck). Keep your shoulders relaxed and feet grounded.

3. Pause for a moment.

4. Inhale, lengthen back to the starting position by lifting the chest forward and not pulling with the arms.

5. Repeat 4–6 repetitions.

Modification: If hip flexors grip or cramp, widen the feet to shoulder-distance or bring the feet closer to the body.

Primary Muscles Worked: Core (rectus and transversus abdominus; internal/external obliques) muscles.

*Spine Twist*:

1. Sit tall with legs together extended straight in front of you, feet flexed, arms extended out to the sides at shoulder height, palms facing down, neutral spine. Your body should form a “T” shape.

2. Inhale, lengthen your spine, reaching the crown of your head toward the ceiling.

3. Exhale, gently twist your torso to the right, keeping your hips stable. Focus on the twist coming from the ribcage and upper back rather than forcing the movement with the arms. If comfortable, pulse three times in the twist position to deepen the stretch. Coordinate your breath and the twist – exhale, twist, exhale, twist, exhale twist.

4. Inhale, return to center.

5. Repeat alternating sides 5 times each.

Modification: If the standard seated position is uncomfortable or causes the back to round, sit cross-legged or with the knees slightly bent. Using a cushion can help lift the hips, making it easier to keep the spine tall and neutral.

Primary Muscles Worked: Core (internal/external obliques and transversus abdominus) and erector spinae muscles.

*Modified Plank*:

1. Begin on all fours on the mat with hands directly under the shoulders and knees directly under the hips. Lower onto the forearms aligning your elbows underneath the shoulders. The hands can be clasped together or rest flat on the mat, whichever feels more stable and comfortable. Walk your knees backward slightly until your body forms a straight line from your knees to your shoulders. Place a folded towel under the elbows/knees if you experience any discomfort when bearing weight on your joints.

2. Exhale, engage your abdominal muscles by drawing your navel in toward your spine to stabilize your torso and protect your lower back. Gaze down at the mat between your hands to keep your head and neck in a neutral position. Squeeze your glutes gently for added stability.

3. Hold the position for 15–20 seconds breathing deeply and smoothly while maintaining your form.

4. Inhale, while still in the position.

5. Exhale, shift your hips back slightly toward your heels about an inch or two.

6. Inhale, come off your forearms one at a time and place your hands under your shoulders.

7. Exhale, shift forward to the starting position.

Primary Muscles Worked: Core (rectus and transversus abdominus; internal/external obliques), erector spinae, latissimus dorsi, deltoids, pectorals, serratus anterior, gluteus maximus, and quadriceps muscles.

**Flexibility and Cool Down (5 Minutes)**

*Cat/Cow*:

1. Begin on all fours on the mat with hands directly under the shoulders and knees directly under the hips. Place a folded towel under the hands/knees if you experience any discomfort when bearing weight on your joints.

2. Exhale, round the back by tucking the tailbone under and tucking the chin toward the chest, casting the eyes down (Cat pose).

3. Pause for a moment.

4. Exhale, gently arch your back by releasing the tailbone upward and letting the chest glide forward between the arms while casting the eyes up (Cow pose). Focus on opening the chest.

5. Pause for a moment.

6. Repeat 4–5 times lengthening (extending) and rounding (flexing) the spine. Feel the spine articulating one vertebra at a time without pain or discomfort.

Note: For the following, it is recommended to sit on a pillow or folded blanket to elevate the hips and alleviate discomfort if the seated position causes any pressure.

*Chest Stretch*:

1. Sit tall with legs crossed or with legs stretched out, hands clasped behind you.

2. Gently push your chest forward and roll your shoulders back and down until you feel a stretch across your chest.

3. Hold for 20–30 seconds, breathing deeply throughout the stretch.

Modification: If shoulder mobility limits putting the arms behind the torso, raise the arms to a goalpost/cactus position (elbows bent at a 90-degree angle, upper arms at shoulder height). Gently draw elbows slightly back, opening the collarbones.

Primary Muscles Stretched: Pectorals, anterior deltoid, biceps brachii, and coracobrachialis muscles.

*Side Stretch*:

1. Sit tall with legs crossed or with legs stretched out. Place your left hand on the floor next to you; it can be near or far.

2. Inhale, raise your right arm up.

3. Exhale, reach your right arm to your left - stretching sideways. Keep sit bones anchored to the floor. Avoid overstretching.

4. Hold the side stretch for a count of three, breathing throughout the stretch.

5. Return to the starting position.

6. Repeat 3–5 times, then switch sides.

Primary Muscles Stretched: Latissimus dorsi, external/internal obliques, quadratus lumborum, intercostal muscles, serratus anterior, and erector spinae muscles.

*Hamstring Stretch*:

1. Sit on the floor with your right leg extended straight in front of you. Bend your left leg comfortably, placing the foot near your inner thigh or farther forward. Place hands either on the floor beside your extended leg or rest them on your thigh.

2. With a lengthened back, hinge slightly forward from your hips and reach toward your foot until you feel a gentle stretch along the back of your thigh. Imagine your torso moving outward over your extended leg rather than downward. Keep your chest up and shoulders back to maintain a neutral spine throughout the stretch.

3. Hold for 20–30 seconds, breathing deeply throughout the stretch.

4. Repeat the stretch 2–3 times, then perform with the left leg.

Primary Muscles Stretched: Hamstrings, gluteus maximus, adductor magnus, gastrocnemius, and erector spinae muscles.

*Shoulder Roll*:

1. Sit tall with legs crossed or with legs stretched out, arms at your sides, elbows soft.

2. Slowly lift your shoulders to your ears, roll them back and squeeze shoulder blades, drop and relax shoulders, then roll forward to finish the circle. Keep your breath slow, steady, and relaxed.

3. Do 5 backward rolls then reverse direction.

*Breathing and Relaxation*:

1. Sit tall with legs crossed or with legs stretched out, hands on thighs. Allow your body to settle into a relaxed position.

2. Inhale slowly through your nose, feeling your abdomen rise and your chest remain still.

3. Exhale slowly through pursed lips, gently drawing your navel toward your spine.

4. Continue to cycle through the inhale and exhale for 20–30 seconds. Focus on relaxing your jaw, neck, and shoulders to assist proper breathing.

## 4. Conclusions

Evidence suggests that Pilates training may represent a feasible and potentially beneficial exercise strategy for individuals with excess body weight and adiposity, with possible positive effects on physical fitness and certain metabolic and mental health outcomes. However, given the methodological limitations, heterogeneity, and restricted generalizability of the available studies, these conclusions remain preliminary. The most recent research findings indicate that supervised Pilates training has the potential to serve as a component of a customized program design for overweight or obese populations, offering a pleasurable and pain-free training alternative in practical settings. In future studies, researchers should emphasize the ideal implementation of training parameters when formulating effective Pilates real-world training protocols for individuals with larger bodies.

## Use of AI tools declaration

The authors declare they have not used Artificial Intelligence (AI) tools in the creation of this article.
